# Trans-Vessel Wall Cell Transplantation, Engraftment, and Tumor Access in the VX2 Rabbit Model

**DOI:** 10.1177/09636897251313678

**Published:** 2025-01-27

**Authors:** Victoria Lövljung, Mathias Waldén, Mikael Sandell, Peter Damberg, Staffan Holmin, Fabian Arnberg Sandor

**Affiliations:** 1 Department of Clinical Neuroscience, Karolinska Institutet, Stockholm, Sweden; 2 Department of Neuroradiology, Karolinska University Hospital, Stockholm, Sweden; 3 Division of Micro and Nanosystems, KTH Royal Institute of Technology, Stockholm, Sweden; 4 MedTechLabs, Stockholm, Sweden; 5 Karolinska Experimental Research and Imaging Centre, Karolinska University Hospital, Stockholm, Sweden

**Keywords:** cancer, cell transplantation, endovascular intervention, endovascular cell transplantation, VX 2, trans-vessel wall device, large animal tumor model, tumor access

## Abstract

The trans-vessel wall device (TW-device) is a new endovascular tool for precise and safe delivery of various payloads (cells, viral, modified RNA, chemotherapy, growth factors) in oncology and regenerative medicine. The twofold aim of this study was to assess cell engraftment and tumor growth using the TW-device for endovascular transplantation and to evaluate its ability to directly access solid tumors. We used the VX2 model in the rabbit kidney to compare percutaneously implanted fresh VX2 cells with TW-device injections of cryopreserved VX2 cells. We demonstrated the feasibility of endovascular transplantation (*n* = 7) of tumor cells, achieving a 57.1% engraftment rate despite cryopreservation, comparable with 70% for percutaneous delivery of fresh cells (*n* = 10). Re-access using the TW-device was 100% successful (*n* = 11) with super-selective intratumoral contrast administration without complications. In conclusion, endovascular transplantation of VX2 cells using the TW-device resulted in proliferating cell grafts in the rabbit kidney establishing functional proof that cells indeed survive handling, preparation, and device passage. We also show the TW-device is able to access solid tumor parenchyma allowing precise intraparenchymal administration.This proof-of-concept study open up possibilities for repeated direct parenchymal injections via the endovascular route in any hard to reach organ.

## Graphical abstract

**Graphical Abstract:**
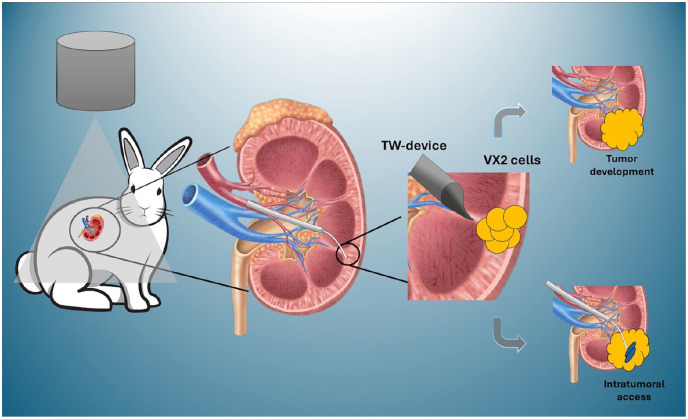

## Introduction

Minimally invasive techniques have revolutionized the field of medicine, allowing physicians to diagnose and treat various medical conditions with increased precision and safety^1^. They are becoming increasingly important in the clinical setting, and there is a growing reliance on these methods with the hope that they can solve several medical problems, especially in the field of oncology and regenerative medicine^2^. One novel route of organ access is the trans-vessel wall (TW) technique by which a novel device (TW-device, commersial version: Extroducer™) is navigated endovascularly to the target organ where the device exits the vessel for delivery of therapeutic agents directly to the target tissue^3^. This route of administration has in several experimental models proven to be safe, and that the TW-device delivery results in higher concentration at the target site and better uptake of cells and medical agents^4,5,6^. Utilization of the TW-device has been demonstrated to be uncomplicated in terms of bleeding and thromboembolic events^3,4^.

Cell therapy is a rapidly advancing field of regenerative medicine showing promise in treating various diseases and conditions, including cancer^7^. The optimal method for delivering therapeutic agents and cell therapy remains a topic of debate, as the most effective approach depends on multifactorial considerations^8^.

Delivering cells directly to target tissue using a TW-device has the potential to enhance engraftment and therapeutic effects of cell transplantation. The TW-device, with a length of at least 100 cm and an inner diameter of 0.147 mm, can effectively deliver cell suspensions to tissue^9^. This has been shown by radioligand cell labeling and by *ex vivo* analyses by immunohistochemistry staining showing presence and proliferation of transplanted cells^5,6^. These methods are, however, indirect and not always robust for determining whether cells are viable and long-term functional in the recipient^10^. To know to what extent cells tolerate procedures used in TW-device cell injection, an experimental model assessing cell viability and function after transplantation is mandated. To robustly explore cell viability and to form a solid tumor target for the TW-device, the VX2 tumor model was chosen as it is a well-studied model for autografting cells forming solid tumors in the rabbit^11^. The induction of tumor via the endovascular route with the TW-device in certain organs would be minimally invasive and would also enable efficient evaluation of novel minimally invasive techniques for tumor treatment^12^.

The primary objective of this study was thus to assess engraftment following endovascular transplantation using the trans-vessel wall technique. To achieve this, we compared percutaneous delivery of fresh VX2 cells to rabbit kidney with endovascular TW-device transplantation of cryopreserved and thawed VX2 cells. To provide flexibility in experimental design, thawed cell suspension was chosen instead of fresh cells for inducing the VX2 tumor in rabbit kidney. The utilization of thawed cells would further highlight the potential of the TW-device, as freezing and thawing processes can potentially cause cellular damage and compromise cellular integrity^13^. Thus, the successful development of a tumor in the recipient would serve as a reliable indication that the cell graft withstood the handling and injection procedures, for example, cryopreservation and thawing followed by the TW-device transplantation technique. In addition, this study aimed to investigate the effectiveness of direct access to tumor parenchyma using the TW-device.

The development of new and effective minimally invasive techniques for the diagnosis and treatment of cancer is of utmost importance^14^. Cancer remains a significant cause of morbidity and mortality worldwide and there is a pressing need for novel and precise treatment options^15,16^. In this regard, the TW-device has the potential to be a valuable tool in the armamentarium of cancer treatment for targeted and safe delivery of different types of payloads such as chemotherapy, biological payloads, and advanced medicinal therapy products.

## Materials and Methods

### Tumor Transplantation and Tumor Access

#### VX2 tumor cell preparation

VX2 tumor cells were purchased from the National Cancer Institute Division of Cancer treatment Diagnosis and Tumor/Cell line Repository, USA. Cells were thawed in lukewarm water and mixed with methylcellulose medium at a 1:1 ratio before injections in hindlimbs of New Zealand White rabbits. Injected volumes were roughly 1 ml, 1 cm deep into the muscle. No immunosuppression was used. The inoculation site was palpated regularly and ultrasonography was used to confirm tumor growth. Once the tumor had achieved desired size, the rabbit was sedated, euthanized and the tumor was surgically excised. Collected tumor tissue was washed through a strainer using Dulbecco’s Modified Eagle Medium (DMEM) and centrifuged at 1,600 r/min for 8 min. The supernatant was discarded, and the pellet was mixed with methylcellulose medium at a 1:1 volume ratio. The cell suspension was either injected into the hindlimb of a donor rabbit to maintain the cell line, or injected percutaneously into a rabbit kidney using ultrasonography guidance, or placed in 1 ml aliquots in cryogenic tubes and frozen in liquid nitrogen for later use.

#### Percutaneous implantation of VX2 tumor cells

The study was approved by the regional ethics committee for animal research and Karolinska Institute guidelines for experiments on rabbits were applied on all animals. The ARRIVE reporting guidelines were used^17^. All animals were tested for various pathogens, and had verified health and immune status. The percutaneous group was designated as the control group, based on prior studies which have consistently shown tumor growth using this method^11,18^. Five adult female New Zealand White rabbits weighing 3.2 to 4.0 kg (Linköping’s Kaninfarm HB, Sweden) received 0.5 to 1 ml of fresh VX2 cell suspension in both kidneys through an ultrasonography guided percutaneous injection. Sedation was induced by intramuscular injection of 0.5 ml/kg Medetomidine and maintained by 0.5 to 1 ml Propofol-lipuro, 10 mg/ml and 0.2 ml Fentanyl, 50 microgram/ml. Supplemental oxygen was provided by mask. The inoculation site was shaved and cleaned with an alcohol-based agent. A 16 G needle was used to inject the VX2 cell suspension. A total of 10 kidneys, five rabbits, were injected. Animals were stabled at Astrid Fagraeus Laboratory Karolinska Institutet with weekly ultrasonography to assess tumor growth.

#### Endovascular transplantation of VX2 tumor cells

All procedures were carried out in a fully equipped angiography lab at the Karolinska Experimental Research Imaging Center (KERIC) and the acclimatization period was at least 7 days. The sample size was determined by considering the complexity of the experiment, limitations in material and the procedure required to develop the TW-device, the availability of equipment including MRI, and insights gained from previous studies conducted by our group. There were no specific inclusion or exclusion criteria, as the primary outcome measured was simply the presence or absence of tumor growth.

A Philips XD20 angiographic system (Philips Medical Systems, Eindhoven, the Netherlands) was used for fluoroscopy and cone-beam computed tomography (CBCT). Visipaque 270 contrast agent (GE Healthcare) was used for contrast-enhanced imaging.

**Figure 1.**
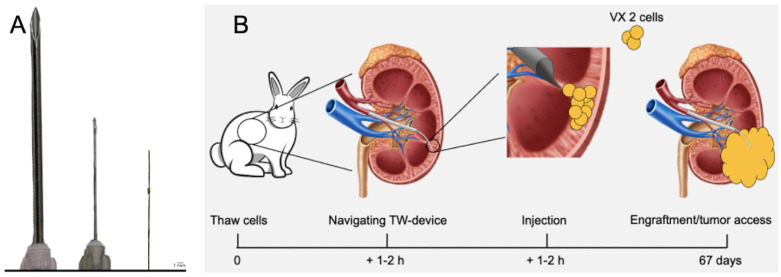
The TW-device compared with other needles and schematic overview of endovascular TW-device cell transplantation. (A) The TW-device (on the far right) shown alongside a 26 gauge (G) needle (in the middle) and an 18G needle (on the far left). Reprinted from Molecular Therapy Methods & Clinical Development, volume 32. Issue 2, 2024, https://doi.org/10.1016/j.omtm.2024.101225. Under a creative commons license: https://creativecommons.org/licenses/by/4.0/. (B) Schematic image of experimental set up in endovascularly transplanted tumors.

Seven adult female New Zealand White rabbits weighing 3.2 to 4.0 kg (Lidköpings Kaninfarm HB, Sweden, Scanbur, United Kingdom, and Envigo, France) were used. All rabbits received 0.5 to 1 ml VX2 cell suspension in one kidney. The number of cells injected ranged from 2.20 × 10⁷ to 6.74 × 10⁷ cells per injection, as detailed in Table 1. Procedures were performed under general anesthesia. Anesthesia was induced by intramuscular injection of 0.5 ml/kg Medetomidine followed by 0.5 to 1 ml Propofol-lipuro, 10 mg/ml and 0.2 ml Fentanyl, 50 microgram/ml. Maintenance of anesthesia was achieved by continuous IV infusion of Propofol-lipuro (10 mg/ml) at a rate of 1.2 ml/kg/h and IV infusion of Fentanyl (50 μg/ml) 0.2 ml/kg/h. A 4F pediatric introducer sheath was inserted into either the right or left femoral artery by ultrasonography guidance, or by surgical exposure of the femoral artery. Next, a 4F catheter (Cobra Hydroglide, Boston Scientific, USA) was navigated to either the left or the right renal artery. A 2.8 F microcatheter (Progreat, Terumo Europe) (Terumo) was then navigated selectively into renal artery divisions entering the renal parenchyma. Next, the TW-device was advanced inside the Progreat microcatheter and then deployed through the arterial wall into the parenchyma. The TW-device is a minimally invasive endovascular tool designed to access target tissues by penetrating the vessel wall. Its catheter system, with a sharp penetrating tip, allows for direct delivery of therapeutic agents or cells to specific organs or tumors. The device’s self-sealing mechanism reduces hemorrhage risks, while its compact size enables targeted interventions with minimal tissue trauma. Studies in organs such as the pancreas, heart, and kidney show that the device can safely deliver therapeutics without the need to seal the penetration site. The TW-device is notably smaller compared with other needles and its compact design enhances safety by minimizing tissue damage during insertion (Fig. 1A)^6,19^. The position of the TW-device-tip in the renal parenchyma was confirmed by CBCT. In select cases, TW-device parenchymal access was confirmed by injecting a small amount of iodine contrast through the TW-device. Next, 0.5 to 1 ml of VX2 cell suspension was slowly infused into renal parenchyma. In all endovascular transplantations VX2 cell suspensions were prepared from frozen aliquots. 1 ml aliquots of VX2 cell suspension in cryogenic tubes stored at −180°C were thawed at 37°C. The cell suspension was dissolved in 3 ml DMEM and centrifuged at 1,600 r/min for 8 min. The pellet was mixed with 1 ml DMEM and kept on ice. A fraction was used for counting cells using a Thermo Fisher Countess II Automated Cell Counter, a quality control step implemented specifically for endovascular transplantations to ensure the viability of thawed cells prior to transplantation. Equal amounts of cell suspension and Trypan Blue Solution, 0.4% (Gibco) were mixed in an Eppendorf Tube using a pipette and administered to an Invitrogen Countess Cell counting Chamber Slide.

**Table 1.** Comparison of Tumor Development and Characteristics Between Percutaneously Implanted and Endovascularly Transplanted Tumors.

	Tumor growth	Cell suspension volume	Cell conc. viable cells	Time to tumor access	Tumor size
	*Yes/no*	*ml*	*per ml*	*Days*	*cm*
Percutaneous					
1	No	1.0	N/A	28	N/A
2	No	1.0		28	N/A
3	Yes	0.5		28	1.0 × 1.5
4	Yes	0.5		28	1.5 × 1.7
5	Yes	0.8		84	2.0 × 2.0
6	Yes, extrarenal	0.8		84	1.5 × 1.8
7	Yes	0.8		83	2.0 × 2.0
8	No	0.8		83	N/A
9	Yes	0.6		89	5.3 × 6.5
10	Yes, extrarenal	1.0		89	4.7 × 4.9
Endovascular					
1	No	1.0	2.20 × 10^7^	72	N/A
2	No	1.0	3.88 × 10^7^	90	N/A
3	Yes	1.0	5.46 × 10^7^	58	2.5 × 2.5
4	Yes	1.0	1.49 × 10^7^	86	1.5 × 2.5
5	Yes	1.0	5.18 × 10^7^	55	4.0 × 2.75
6	No	1.0	6.74 × 10^7^	90	N/A
7	Yes	0.5	6.89 × 10^7^	69	3.0 × 3.0
*P*-value	0.64	0.14	N/A	0.40	0.51

This table presents key study findings comparing percutaneous implantation and endovascular transplantation of VX2 cells. Percutaneous and endovascular groups were assessed for tumor development, time to tumor formation (in days), tumor size (measured in centimeters) and amount of cell suspension injected (in milliliters). The percutaneous group exhibited a tumor development rate of 70%, with an average time to tumor formation of 69 days. The tumors in this group had an average size of 2.74 × 2.36 cm (SD 1.49 × 1.94 cm), and an average of 0.78 ml of cell suspension was injected. In contrast, the endovascular group showed a tumor development rate of 57.1%, with an average time to tumor formation of 67 days. The tumors in this group had an average size of 2.75 × 2.69 cm (SD ±1.12 × 0.24 cm), and an average volume of 0.92 ml of cell suspension was injected. Statistical tests used: Fisher’s exact test for Tumor Growth, Mann–Whitney *U* test for Time to Tumor Access and Tumor Size, and *t* test for Injection Volume.

Prior to infusing the VX2 cell suspension in the kidney it was vortexed and aspirated into a 250 or 500 μl Hamilton 1710, Large hub RN, syringe with a 22G blunt needle, 20 mm. The needle was disconnected, and the Hamilton syringe attached to the TW-device enabling infusion of the cell suspension. This step was repeated until 0.5 to 1 ml was administered into the kidney. After withdrawal of the TW-device an angiogram was obtained assessing renal artery patency. Animals were woken from anesthesia and stabled at an animal care facility where they were observed and examined regularly with ultrasonography to detect and follow tumor growth.

#### Endovascular access and injections in VX2 tumor

All rabbits in both endovascular and percutaneous groups that exhibited tumor growth were subjected to endovascular tumor access (*n* = 11). Animal preparation, anesthesia procedures and endovascular access to the renal artery was performed as described above. A renal artery angiogram was obtained, showing the kidney vasculature and the tumor. A microcatheter (Progreat, Terumo, Japan) was advanced to a renal artery branch adjacent to the tumor. The TW-device was inserted through the microcatheter and advanced through the vessel wall toward the tumor center. The position of the TW-device -tip was confirmed with CBCT. A Hamilton syringe was attached to the TW-device and with its tip in the tumor, 0.5 to 1 ml of iodine –fat emulsion (Lipiodiol, Guerbet) or Visipaque 270 was infused directly into the extra vasal tumor tissue. In some instances, methylene blue was administered in this position to subsequently verify tumor parenchymal access macroscopically. Once all imaging was obtained with the angiographic system, all instruments were withdrawn, and the animal was transferred to the MRI for further imaging.

### Data processing and analysis

#### MRI data acquisition

MRI data were acquired using a 9.4 T MRI scanner. The scanner was controlled with software VnmrJ v4.0 Revision A (Agilent). A gradient insert with an inner diameter of 20 cm was used (Varian, Yarnton, United Kingdom). For RF-excitation an actively tuned quadrature birdcage resonator (Rapid Biomedical, Würzburg, Germany) was used and the signal was received through a four-channel phased array coil, designed for Rabbit brain, (Rapid Biomedical, Germany) which was positioned from the side to have the strongest possible signal from the tumor-bearing kidney.

Following intravenous administration of 0.1 mmol/kg Gadobutrol (Bayer, Germany), the rabbits were euthanized either before or after MRI. Subsequently, the tumor-bearing kidney was imaged using a gradient echo sequence with the following parameters: 52 contiguous slices of 1 mm thickness, matrix 256 × 256, Field-of-View 150 and 128 mm in the read-out and phase-encode directions, respectively, repetition time 1,200 ms, time to echo 6.70 ms. Oblique slice orientation was with the read-out direction in the rostral-caudal direction and the phase-encode direction mainly left-right.

#### Hematoxylin and Eosin staining

The animals were sacrificed using a lethal dose of IV pentobarbital and subjected to nephrectomy. Kidney tissue specimens were frozen on dry ice, cut with cryostat (CM 3000, Leica instruments GmbH, Germany) in 14 to 20 μm sections and stored at −20°C until staining. All sections were stained with hematoxylin and eosin staining according to Mayer’s protocol^20^. Stained slides were assessed using light microscopy, and images were captured using a Canon 7D camera (Canon, Ota City, Japan) attached to the microscope.

#### Statistical analysis

Statistical analysis was performed to assess key variables related to tumor development and injection volume consistency. Fisher’s exact test was used to evaluate the binary outcome of tumor growth (yes/no), while the Mann–Whitney *U* test was applied to continuous outcomes, including time to tumor access and tumor size. In addition, a *t* test was conducted to assess the consistency of injection volumes across the two groups.

## Results

### Endovascularly Transplanted VX2 Cells Were Successful to a Comparable Extent as Percutaneously Implanted Cells

**Figure 2.**
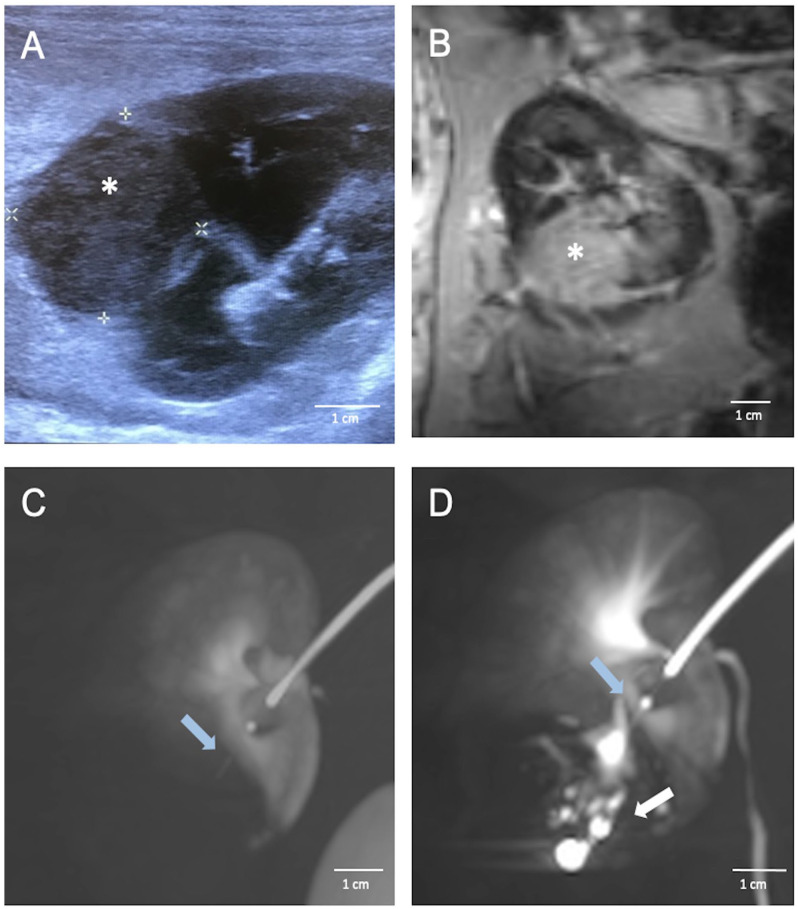
Multimodal imaging assessment of tumor development in rabbit kidney using ultrasonography, contrast-enhanced cone-beam CT, and MRI. (A) Ultrasonography showing a well-defined tumor, indicated by the asterisk, in rabbit kidney after endovascular transplantation of VX2 cells. (B) MRI image of a rabbit kidney with VX2 tumor (asterisk). The MRI scans clearly depict distinct signal abnormalities within the expansive process of the kidney, consistent with the presence of a tumor. (C) A contrast-enhanced cone-beam CT image of a rabbit kidney with a tumor accessed with the TW-device (blue arrow). The image demonstrates the capability of the TW-device to be accurately positioned within the tumor area for targeted interventions. (D) A contrast-enhanced cone-beam CT image showcasing intratumoral position of the TW-device (blue arrow) distributing contrast agent intratumorally (white arrow). To enhance the clarity and detail of the cone-beam CT images, Maximum Intensity Projection (MIP) was employed.

One aim of this study was to investigate the feasibility of endovascular cell transplantation using the TW-device. We used the VX2 tumor model in rabbits for robust functional evaluation of cell engraftment following endovascular injections with the TW-device. The study was conducted with two groups of rabbits, five in the percutaneous group and seven in the endovascular group. The percutaneous group received freshly harvested VX2 cell grafts in both kidneys (*n* = 10), and the endovascular group received cryopreserved cell grafts in one kidney (*n* = 7). All kidneys were injected with 0.5 to 1 ml of cell suspension each, containing between 2.20 × 10⁷ and 6.74 × 10⁷ cells, the precise cell count for each injection is provided in Table 1. No complications, such as bleeding, thrombosis or infection, were observed during procedures. All animals were monitored with ultrasonography to detect and follow tumor growth for a maximum of 90 days or until the tumor reached a size of 4 cm measured in longitudinal axis (Figs. 1B and 2A). Statistical analysis revealed no significant differences between the percutaneous and endovascular groups in tumor growth (*P* = 0.64), time to tumor access (*P* = 0.40), or tumor size (*P* = 0.51). In addition, injection volumes were consistent across both groups (*P* = 0.14).

In percutaneously injected animals, a total of 10 kidneys (five rabbits) were injected with an average of 0.82 ml of fresh VX2 tumor cells. Tumors were successfully grown in seven instances, with five cases occurring in the kidney and two within the perirenal fat, yielding a general engraftment rate of 70%, and 50% in the kidney (Table 1). Ultrasonography failed to detect tumors in the perirenal fat; however, one tumor was visualized using contrast-enhanced CBCT which exhibited an expansive growth outside the kidney. The second tumor was identified during postmortem nephrectomy.

In the endovascularly transplanted group, seven rabbits were successfully transplanted with an average of 0.88 ml of thawed VX2 cells containing an average of 4.76 × 10^7^ live cells and showed no adverse side effects. Out of these, four rabbits showed successful engraftment with a tumor incidence rate of 57.1% (Table 1). No cases of extrarenal growth occurred in this group.

In the percutaneous group, the mean tumor size in the longitudinal axis was 2.74 × 2.36 cm (SD 1.49 × 1.94 cm) at an average of 69 days (SD 28.78 days) after injection (Table 1). In the endovascular group, the mean tumor size in the longitudinal axis was 2.75 × 2.69 cm (SD ±1.12 × 0.24 cm) at an average of 67 days (SD ±14.02) after transplantation. There was no statistical difference between the groups, as determined by statistical analysis using Fisher’s exact test (*P* = 0.64) and *t* test (*P* = 0.29).

### Endovascular Intratumoral Access Using the TW-Device Was Successful in All Instances

**Figure 3.**
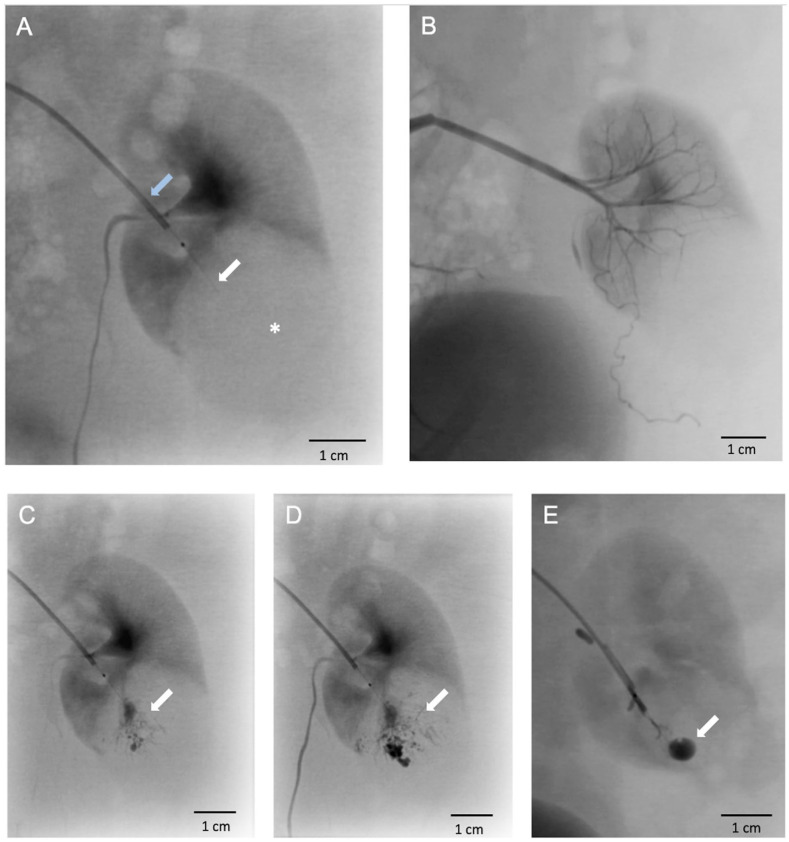
X-ray of rabbit kidney showing trans-vessel wall device access and tumor-selective Lipiodol delivery. (A) X-Ray of a rabbit kidney harboring a VX2 tumor indicated by the asterisk. A 4F catheter (blue arrow) is placed in a renal segmental artery guiding the trans-vessel wall device (white arrow) in the tumor parenchyma. (B) Tortuous vessels exhibiting modest contrast enhancement. (C–E) Injection of Lipiodol (Guerbet, France) with diffusion within the tumor parenchyma is seen (white arrows).

**Figure 4.**
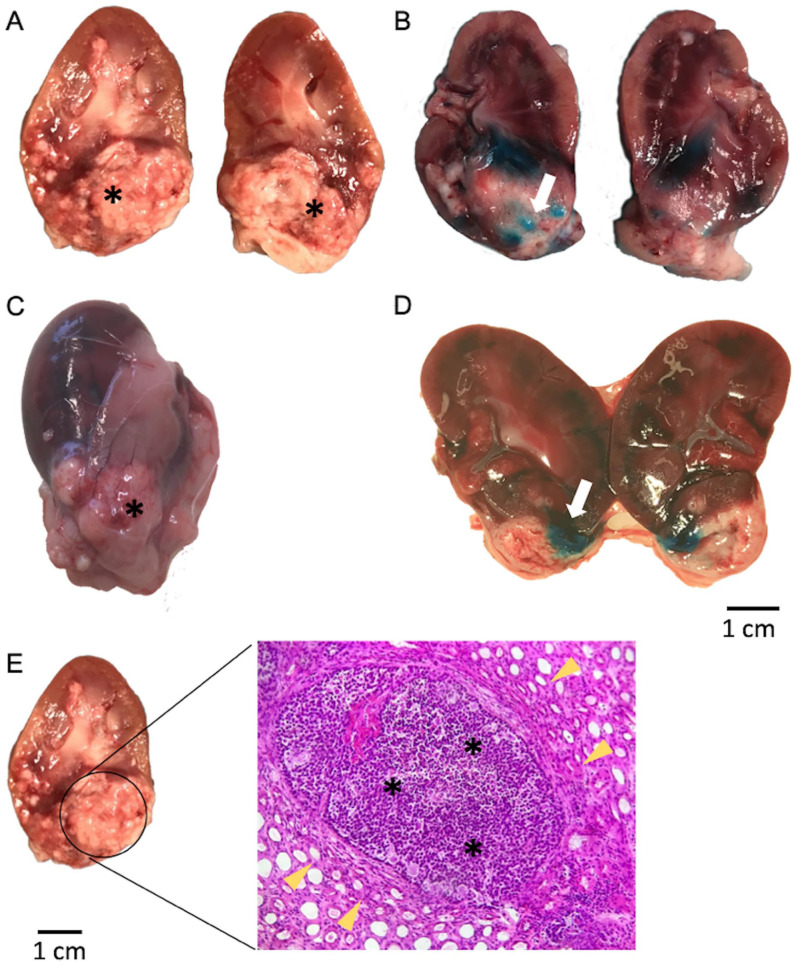
Macroscopic and histological analysis of tumor-bearing rabbit kidney: methylene blue distribution via trans-vessel wall device and hematoxylin and eosin staining. (A) Macroscopic view of an excised kidney bearing a tumor that was endovascularly transplanted. The tumor location, marked by an asterisk, corresponds to the site where the trans-vessel wall device was positioned during the injection of VX2 cells. This confirms the viability and engraftment following the endovascular transplantation of cells using the TW-device. (B) Shows methylene blue (white arrow) successfully administered to the tumor using the TW-device, proving its capability to deliver agents to tumor tissue. (C) Percutaneously implanted VX2 tumor (asterisk) to the inferior pole. (D) Percutaneously implanted VX2 tumor to the inferior pole with successful methylene blue (white arrow) administration using the TW-device. (E) Stained kidney sections, revealing distinct cellular features. The high nucleus-to-cytoplasm ratio and few specialized structures indicate increased cell proliferation and tumor growth (asterisk). Adjacent normal kidney tissue (yellow arrowheads) serves as a reference for comparison. The staining results further support the presence of a tumor in the kidney and provide visual evidence of the cellular changes associated with tumorigenesis.

Once the tumor developed, attempts were made to achieve endovascular intratumoral access. The vascular anatomy of the rabbit kidney is similar to that of humans with the renal artery arising from the abdominal aorta branching into interlobar arteries entering the renal hila^21^. Renal angiography of tumor-bearing kidneys revealed a subtle contrast-enhanced spherical expansion in contrast to the uptake pattern within the surrounding renal tissue (Fig. 3A). No discernible arterial supply branch from the kidney directed toward the tumor was evident. Delicate, tortuous vessels, exhibiting modest contrast enhancement, were observed converging toward the tumor from extrarenal regions, suggesting a plausible vascular supply originating from the perirenal fat (Fig. 3B). The procedure was challenged by the small vessel diameters and the tissue resistance of the solid tumor components. Otherwise, accessing the tumor was uncomplicated and the maneuver was straightforward and successful in all attempts. With the TW-device in place in the tumor, Lipiodol was injected under fluoroscopy (Figs. 2D and 3C–E). CBCT images were obtained showing the exact position of the TW-device and distribution of Lipiodol in the tumor (Fig. 2C, D). Further on, methylene blue, a less viscous fluid, was administered for necropsy evaluation and more readily diffused throughout the tumor (Fig. 4B, D). After withdrawal of the TW-device, angiography runs were normal showing no contrast extravasation or other manifests of vascular injury. MRI scans showed distinct signal abnormalities in the expansive process of the kidney consistent with tumor parenchyma (Fig. 2B). No expansive hematoma or other injury was observed.

### Tumor Engraftment Was Confirmed by Macroscopic Evaluation and Histological Analysis

After completion of imaging, animals were sacrificed, and the kidney was surgically excised. A gross examination of the excised kidney revealed a well-circumscribed, slightly coarsely nodular mass representing the cancer tumor at the site of the injection site (Fig. 4A, C). The tumor tissue appeared grayish-white in contrast to the surrounding healthy kidney parenchyma. Upon slicing the kidney, the site of injection was shown by the methylene blue deposition delivered using the TW-device (Fig. 4B, D). These results demonstrate the feasibility of endovascular trans-vessel wall access to tumor parenchyma via the TW-device, creating a working channel to the tumor that allows targeted intra and/or peritumor administration of different types of payloads, including cell therapies.

Samples for histological analyses were taken for further evidence of tumor growth. The stained samples were observed under a microscope showing prominent nucleoli, a high nucleus-to-cytoplasm ratio, many mitoses, and relatively few specialized structures demonstrating typical growth pattern for a carcinoma (Fig. 4E).

## Discussion

This study explores cell engraftment following endovascular transplantation using the trans-vessel wall technique. Engraftment was assessed by comparing outcomes of percutaneously injected fresh VX2 cells with endovascularly transplanted cryopreserved VX2 cells. Herein, we demonstrate that endovascular transplantation of VX2 cells to the kidney in rabbits is a feasible technique resulting in functional proliferation of transplanted cells. We also show that it is possible to re-access the tumor and/or peritumor region with this endovascular method for targeted delivery of different types of payloads such as cells, modified RNA, oncolytic viruses or antibodies^5,6,22^.

The higher success rate of tumor growth in the percutaneous group is likely attributed to the use of fresh cells, which typically exhibit greater metabolic activity and proliferative potential compared with cryopreserved cells^13,23^. Also, the freeze-thaw process can compromise cellular function and viability, further complicating tumor growth^24,25^. While including both fresh and cryopreserved cells across percutaneous and endovascular groups would offer more insight into the impact of cell preparation, this study focused on testing the TW-device with cryopreserved cells due to their reduced viability and proliferative capacity after freezing and thawing. This choice allowed for a more rigorous test of the TW-device’s ability to maintain cell function under demanding conditions. Testing fresh cells with the TW-device would have been an easier approach. If cryopreserved cells had been used in the percutaneous group—a more established technique—a comparison with fresh cells would have been more necessary to isolate the impact of cryopreservation. The experimental design, using fresh cells in the percutaneous group and cryopreserved cells with the TW-device, minimized logistical complications and provided a more stringent test of the TW-device. In addition, this approach was motivated by practical considerations, such as reducing the overall duration of the experiment and minimizing anesthesia time. Future studies will include both fresh and cryopreserved cells across all delivery methods for a more comprehensive comparison. An essential quality control step was implemented for the endovascular transplantations, where cells were counted after thawing to confirm their viability before transplantation. Given that these cells had undergone freezing and thawing, ensuring their viability was crucial. Although the tumor growth rate was faster in the percutaneous group, this was expected, given the inherently greater viability and proliferative potential of fresh cells^26^.

While the TW-device successfully delivered VX2 cells and facilitated tumor engraftment in the VX2 rabbit model, several factors, other than cryopreservation, may have contributed to unsuccessful engraftments. Handling during delivery through the TW-device could have further compromised cell viability. While the TW-device is minimally invasive, the mechanical passage of cells through the device may expose them to shear forces, especially for cryopreserved cells already under stress, leading to lower engraftment rates. Other factors that could have influenced engraftment include the immunogenicity of VX2 tumor cells^11,27^. While the VX2 model is well-established for tumor induction, it is possible that immune responses in the recipient animal contributed to graft rejection, although no significant immune responses were observed. Biological variability among individual animals also plays a role. Differences in immune responses, tissue environment, or metabolic processes could affect engraftment success, even under controlled conditions^28^. The inherent variability in the VX2 model, reported in previous studies, may have also contributed to our results^11^. Finally, the handling and preparation of cells before injection introduce potential risks for cell viability^29^. Despite efforts to minimize handling time and ensure cell quality through rigorous checks, these factors remain an inevitable part of transplantation protocols.

Generally, in cell transplantation, the viability of graft transplants poses a significant challenge as well as limitations associated with existing modalities of cell delivery^30^. One key limitation is the substantial cell loss that occurs shortly after transplantation, irrespective of cell type, formulation, delivery technique, or disease state^31^. Also, the precision required for delivering cell-based therapeutics is notably higher compared with conventional therapeutics. Needle-based delivery to dense tissues, as in the percutaneous group in this study, may result in significant backpressure, leading to displacement of cell suspension retrogradely through the injection track after needle removal^32^. This was observed in two animals in the percutaneous group, with tumors developing in the injection track in the perirenal fat. No such events occurred in the endovascular group, enhancing the minimally invasive nature and precise delivery capabilities of the TW-device—a compact system with auto-sealing properties due to the extremely small outer diameter, that reduces backflow.

By implementing this experimental model, we gained a comprehensive understanding of the ability of cells to withstand the TW-device’s injection procedures while maintaining their viability and functionality. Although the device’s narrow inner diameter and length could potentially exert harmful pressures and shear stress on cells, leading to engraftment failure, our results showed that cryopreserved and thawed cells tolerated injection through the TW-device, producing viable grafts and developing tumors. The VX2 model, with its use of clinical routine materials and transplantation without immunosuppression, has several advantages. However, tumor cells are highly persistent, and their resilience may limit broader conclusions^33^, highlighting the need for further experiments using therapeutic cells to evaluate the applicability of this technique in cell-based therapies. Tumor cells were chosen in this study to enable autologous transplantation without the need for immunosuppression. Recent studies have shown that mesenchymal cells transplanted with the TW-device survive and are functional for protein production^6^. In addition, insulin-producing cells labeled with indium-11 demonstrated uptake in the pancreas detected by single-photon emission CT^5^. These findings support the use of the TW-device for transplanting functional cells, with this study serving as proof of concept for long-term cell survival. In contrast to tumor cells, therapeutic cells like T cells or mesenchymal stem cells (MSCs) would require different assessment methods for viability, such as imaging or biomarker assays. This study, using tumor cells, provided a direct indicator of cell viability through observable tumor growth. Future studies will explore the use of therapeutic cell types for regenerative and immunotherapeutic applications, further validating the TW-device’s versatility across diverse therapeutic contexts.

The study also aimed to examine the efficacy of direct tumor access using the TW-device. We successfully accessed the tumor using the TW-device, enabling a super-selective injection of lipiodol and methylene blue as proof of concept. Tumor microvessels are anatomically dilated, tortuous, irregular, and display a disorganized branching pattern, differing from the organized branching of normal tissue^34^. Indeed, when accessing the kidney artery selectively, evident tumor feeding arteries for catheterization were notably absent in both endovascularly transplanted and percutaneously implanted tumors. The vascularity exhibited a rather diffuse distribution. Despite this vascular intricacy, we successfully achieved intratumoral access utilizing the trans-vessel wall technique from normally appearing small arteries adjacent to the tumor itself. This technique facilitated super-selective access, allowing for precise intervention despite the absence of clearly delineated supplying vessels, previously also shown in other hard-to-reach-areas, such as brain and liver^35,36^.

The optimal approach for delivering therapeutic agents and cells in clinical practice remains a subject of ongoing debate and advancement. Various delivery methods, such as intravenous injection, intra-arterial delivery, and localized administration via catheters or implantable devices, are being explored to determine the most effective approach^37,38^. For cell-based therapies, conventional intravenous injection often results in cells becoming trapped in the lungs, spleen, and liver^32,38^. Direct delivery to the target tissue through endovascular (intra-arterial) or direct tissue (intramuscular and intraparenchymal) injections has shown potential in enhancing engraftment and therapeutic effects^35^.It would also make it possible to reduce the total administered dose of any payload with potential reduction of side effects and also significantly reduced cost.

In summary, challenges associated with cell-based therapies and biological therapies such as modified RNA, oncolytic viruses and gene therapies require innovative delivery techniques to optimize cell survival, local targeting and full effect without causing systemic side effects and too high costs. The TW-device presents a novel approach for targeted delivery directly to any organ or tumor via the endovascular route and have numerous potential applications both in regenerative medicine and oncology. Here, we investigate a clinically relevant scenario by proving long-term cell viability of cells after passage through the delivery system followed by direct access to the tumor with the TW-device for delivery of any therapeutic payload. This study highlights that successful implementation of the TW-device may be a highly interesting option for targeted delivery to organs that are difficult or risky to reach with conventional methods.

## Supplemental Material

sj-docx-1-cll-10.1177_09636897251313678 – Supplemental material for Trans-Vessel Wall Cell Transplantation, Engraftment, and Tumor Access in the VX2 Rabbit Model Supplemental material, sj-docx-1-cll-10.1177_09636897251313678 for Trans-Vessel Wall Cell Transplantation, Engraftment, and Tumor Access in the VX2 Rabbit Model by Victoria Lövljung, Mathias Waldén, Mikael Sandell, Peter Damberg, Staffan Holmin and Fabian Arnberg Sandor in Cell Transplantation
